# Physiological Translocation of Lactic Acid Bacteria during Pregnancy Contributes to the Composition of the Milk Microbiota in Mice

**DOI:** 10.3390/nu10010014

**Published:** 2017-12-23

**Authors:** Javier de Andrés, Esther Jiménez, Isabel Chico-Calero, Manuel Fresno, Leónides Fernández, Juan Miguel Rodríguez

**Affiliations:** 1 Department of Nutrition, Food Science and Food Technology, Complutense University of Madrid, 28040 Madrid, Spain; javierdeandres.vet@gmail.com (J.d.A.); leonides@ucm.es (L.F.); 2 Centro de Biología Molecular Severo Ochoa, Consejo Superior de Investigaciones Científicas (CSIC), Universidad Autónoma de Madrid (UAM), and Instituto Sanitario de Investigación Princesa, 28049 Madrid, Spain; iccalero@gmail.com (I.C.-C.); mfresno@cbm.uam.es (M.F.)

**Keywords:** human milk, translocation, *Lactobacillus salivarius*, *lux*, bioluminescence, pregnancy, lactation

## Abstract

The human milk microbiota is a complex and diverse ecosystem that seems to play a relevant role in the mother-to-infant transmission of microorganisms during early life. Bacteria present in human milk may arise from different sources, and recent studies suggest that at least some of them may be originally present in the maternal digestive tract and may reach the mammary gland through an endogenous route during pregnancy and lactation. The objective of this work was to elucidate whether some lactic acid bacteria are able to translocate and colonize the mammary gland and milk. For this purpose, two lactic acid bacteria strains (*Lactococcus lactis* MG1614 and *Lactobacillus salivarius* PS2) were transformed with a plasmid containing the *lux* genes; subsequently, the transformed strains were orally administered to pregnant mice. The murine model allowed the visualization, isolation, and Polymerase Chain Reaction (PCR)-detection of the transformed bacteria in different body locations, including mammary tissue and milk, reinforcing the hypothesis that physiological translocation of maternal bacteria during pregnancy and lactation may contribute to the composition of the mammary and milk microbiota.

## 1. Introduction

Human milk contains microorganisms that, together with other milk components, play a pivotal role in the early colonization of the infant gut. The use of diverse culture-dependent or-independent techniques has allowed either the isolation or detection of a wide spectrum of commensal bacteria in milk samples provided by healthy women worldwide [1]. Most research efforts have been focused on lactobacilli and bifidobacterial isolates because of their potential to be used as probiotics [2].

Traditionally, the presence of bacteria in human milk was related to contamination from the mother’s skin or infant’s oral cavity [2]. In addition, bacterial translocation from the digestive tract of healthy women has been proposed as a source of bacteria for the mammary gland during late pregnancy and lactation [3]. In fact, physiological translocation of commensal bacteria from the gut to different mucosal surfaces has already been shown in vitro and in vivo through a mechanism involving complex interactions between bacteria, epithelial cells, and immune cells (including dendritic cells and macrophages) [3,4]. Previous studies have reported that oral administration of some lactobacilli strains to lactating women leads to their presence in milk [5,6]. However, more mechanistic studies focused on the potential bacterial transfer from the gut to the mammary glands of healthy hosts are required to further confirm such findings.

Bioluminescent whole-body imaging allows rapid and real-time monitoring of bacteria in vivo. This system captures photons of light emitted by naturally luminescent bacteria or by those that have been genetically manipulated to produce bioluminescence. The bioluminescence reaction involves a luciferase-catalyzed intracellular oxidation of a long-chain fatty aldehyde (R-CHO) together with a concomitant reduction of flavin mononucleotide (FMNH_2_). Such reactions lead to the generation of blue-green light, as follows: FMNH_2_ + O_2_ + R-CHO → FMN + H_2_O + R-COOH + Light (~495 nm). The *lux* operon (*luxABCDE*) contains the genetic determinants for bioluminescence and includes the structural genes (*luxA* and *luxB*) coding for the two subunits (α and β, respectively) of the luciferase enzyme as well as three additional genes (*luxC*, *luxD*, and *luxE*) encoding the fatty acid reductase complex responsible for fatty aldehyde synthesis [7].

Bioluminescence imaging has successfully demonstrated in vivo translocation of pathogenic strains of *Escherichia coli* and *Citrobacter rodentium* in mice [8,9,10], and persistence of *Lactococcus lactis* and *Lactobacillus plantarum* strains in the murine gastrointestinal tract [11]. In addition, translocation of bioluminescent bifidobacteria from the gastrointestinal tract of mice, and their subsequent selective recruitment by tumoral cells, have been shown using such an approach [12]. In this context, the objective of this work was to obtain bioluminescent lactic acid bacteria (LAB) strains to provide in vivo evidence of their physiological translocation in mice during late pregnancy and lactation.

## 2. Material and Methods

### 2.1. Bacterial Strains and Media

The LAB strains used in this work as recipients for the *lux* genes were *Lactococcuslactis*MG1614, a laboratorial strain widely used in studies dealing with molecular biology of lactococci [13], and *Lactobacillus salivarius* PS2, a probiotic strain originally isolated from human milk [14,15,16]. *L. lactis*MG1614 was routinely grown in M17 (Oxoid, Basingstoke, UK) supplemented with 0.5% (*w*/*v*) glucose (GM17 medium) and incubated at 30 °C, while *L. salivarius*PS2 was grown in Man, Rogosa, and Sharpe (MRS) (Oxoid) medium and incubated at 37 °C. Competent *Escherichia coli* cells were purchased from Bioline (BIO-85027; Bioline Reagents Ltd., London, UK). *E. coli* was grown in Luria Bertani (LB) medium and incubated at 37 °C. When required, erythromycin (Em) (Sigma-Aldrich, St. Louis, MI, USA) was added to the cultures at the following concentrations: 150 μgmL^−1^ for *E. coli*, 2.5 μg mL^−1^ for *L. lactis*MG1614, and 5 μg mL^−1^ for *L. salivarius* PS2. Previously, the Em resistance of *L. salivarius* PS2 had been tested in MRS broth containing Em concentrations ranging from 0.25 to 5 μg mL^−^¹ at 37 °C for 24 h and bacterial growth was detected by measuring the OD_600_ of the cultures.

### 2.2. Construction of pMG36e::luxAB and pMG36e::luxABCDE

Plasmid pXen-5 (Xenogen Bioware, Alameda, CA, USA) was used as template to amplify the *luxABCDE* operon using primers XAF1/XBR1 (which generate a DNA fragment containing only the structural genes *luxAB*) and XAF1/XER2 (which generate a DNA fragment containing the complete *luxABCDE* operon) [17]. However, primers XAF1/XBR1 were modified to add restriction sites *Sac*I/*Sma*I and *Sac*I/*Sal*I, respectively, at the ends of the amplicons (Table 1). PCR was performed using Phusion Hot Start II DNA High-Fidelity DNA Polymerase (Finnzymes, Vantaa, Finland) with the following conditions: 98 °C for 30 s; 35 cycles at 98 °C for 10 s, 72 °C for 1 min, 72 °C for 5 s; and finally, 72 °C for 5 min. PCR products were purified using Sure Clean Plus (Bioline, London, UK) following the manufacturer’s instructions. 

After digestion of the vector (plasmid pMG36e) with the corresponding enzymes (New England Biolabs. *Sma*I, at 25 °C; *Sac*I and *Sal*I at 37 °C; all incubations occurred overnight), ligations were performed overnight at 16 °C with T4 DNA ligase (Roche, Mannheim, Germany) to generate plasmids pMG36e::*luxAB* and pMG36e::*luxABCDE*, respectively. Plasmid pMG36e is a 3.6 kb expression vector carrying an Em resistance gene and the strong P32 promoter [18]. Both plasmids were separately introduced into competent cells of *E. coli* (BIO-85027; Bioline), following the manufacturer’s instructions. Cells were plated on LB plates supplemented with Em (150 μg mL^−1^) in order to select for transformants. 

### 2.3. Transformation of L. lactis MG1614 and L. salivarius PS2

Both plasmids were extracted from transformed *E. coli* cells using the Qiagen plasmid mini kit (Qiagen, Hilden, Germany) and electroporated into *L. lactis*MG1614 cells as described previously [19]. After electroporation, *L. lactis* cells were plated on GM17 supplemented with Em (2.5 μg mL^−1^) and incubated at 30 °C for 48 h. Subsequently, both plasmids (containing P32-*luxAB* and P32-*luxABCDE*, respectively) were isolated from the previously transformed *L. lactis* MG1614 cells and electroporated into competent *L. salivarius*PS2 cells, which had been obtained as previously described [20]. Then, bacterial cells were plated on MRS plates supplemented with Em (5 μg mL^−1^) and incubated at 37 °C for 48 h. The stability of the recombinant plasmids was assayed by daily sub-culturing of the recombinant strains in non-selective media during seven consecutive days. Aliquots of each subculture were plated on selective (Em-supplemented MRS) and non-selective (MRS) agar plates [21]. Plasmid maintenance was determined by comparing the numbers of colonies that grew on both types of media.

Presence of the plasmids in the transformant cells was confirmed by PCR. Primers lux1280F/lux1732R allow the amplification of a 452 bp fragment located between *luxA* and *luxB* genes, while primers lux4807F/lux5068R were designed to amplify a 261 bp fragment between *luxD* and *luxE* genes (Table 1). PCR was performed using My Taq™ Red DNA Polymerase (Bioline) and the following conditions: 94 °C for 4 min; 25 cycles at 94 °C for 30 s, 53 °C for 30 s, 72 °C for 30 s, and 72 °C for 5 min. The amplified fragments were visualized by electrophoresis in a 1.2% agarose gel after 90 min at 90 V.

### 2.4. Bioluminescence Assays

Bioluminescence assays were performed in collaboration with the Biolum Lab of the “Red de Laboratorios de la Comunidad de Madrid”. Production of bioluminescence by the bacterial cultures was measured directly in broth medium (1 mL) with a luminometer (Biocounter^®^ M1500 Lumacbv, Landgraaf, The Netherlands), and the bioluminescence was expressed as Relative Light Units (RLUs). The presence of bioluminescent signals was also analyzed on the surface of agar plates using the IVIS 100 imaging system (In Vivo Imaging System, Xenogen, Perkin Elmer, Hopkinton, MA, USA), which consists of a cooled charge-coupled-device (CCD) camera mounted on a light-tight specimen chamber. The signal intensity was quantified as photon counts per second (p/s).

### 2.5. In Vivo Translocation Model

Ten-week-old pregnant Balb/c mice (Day 5 of gestation) were used to assess the potential in vivo translocation of the transformed *L. lactis* and *L. salivarius* strains. Animals were kept in the Animal Facility of Centro de Biología Molecular Severo Ochoa (CBM-CSIC, Madrid, Spain), and housed individually (one pregnant mouse per cage) in 1264C Eurostandard Type II cages (26.7 × 20.7 × 14.0 cm-floor area 370 cm², Tecniplast, Buguggiate, Italy) with bedding, food (Diet 2018, Harlan, Correzzana, Italy) and water available ad libitum, under a temperature (22 °C) and light-controlled (12 h) cycle. All experimental procedures complied to the principles of good laboratory animal care, were carried out in compliance with national legislation following the EU-Directive 2010/63/EU for the protection of animals used for scientific purposes, and were approved by the ethics committee for animal experimentation and the Animal Welfare Body of CBM/Complutense University of Madrid, Spain (Code 15/017E; approval date: January 2015). All adequate measures were taken to minimize animal pain or discomfort.

Bacterial strains were grown overnight in GM17 supplemented with Em (2.5 μg mL^−1^) at 32 °C (*L. lactis* cells) or MRS supplemented with Em (5 μg mL^−1^) at 37 °C (*L. salivarius* cells). Cells were harvested by centrifugation (18,800×*g* for 10 min at 4 °C) and the pellet was resuspended in a mixture of 10% skimmed milk with 10% of 2.5 M sucrose. The mix was dispensed as single-doses (200 µL, ~10^9^ CFU/dose), lyophilized, and kept at −20 °C until their administration to mice. The stability of the doses was assessed weekly by plate count on GM17 (Em: 2.5 μg mL^−1^) or MRS (Em: 5 μg mL^−1^) agar plates.

Test mice (*n* = 6) received a daily dose (at the same time each day) of transformed *L. lactis* (*n* = 2) or *L. salivarius* (*n* = 4; two pregnant and two non-pregnant females) intragastrically via oral gavage until delivery (~20 days). Control mice (*n* = 5) received a daily dose (200 µL) of the probiotic matrix (10% skimmed milk with 10% of 2.5 M sucrose) also by intragastric administration. 

At Day 15 of pregnancy and within the first 6 h after delivery, in vivo bioluminescence imaging of the mice was performed using the multimodal IVIS 100 imaging system described above. For this purpose, mice were anesthetized with 2% isoflurane and placed into the camera chamber, where a controlled flow of 1.5% isoflurane-supplemented air was administered through a gas anesthesia system. Bioluminescence was quantified as p/s using the Living Image^®^ software (Caliper Life Sciences, Waltham, MA, USA).

Within the first 12 h after delivery, the female mice and their offspring (before weaning) were euthanized using a mixture of CO_2_/O_2_ according to the EU guidelines and necropsies performed under sterile conditions. Samples of feces, milk, urine, blood (EDTA tubes), and biopsies of intestine (large and small), stomach, liver, spleen, kidneys, mammary glands, uterus, Peyer’s patches, and mesenteric nodes were collected and stored at 4 °C for microbiological and microscopy-based analysis. 

To evaluate potential translocation of the transformed strains from the gastrointestinal tract to different tissues, the biological samples (biopsies, urine, milk, and feces) were homogenized in peptone water (with the exception of urine and milk) and decimal serial dilutions were spread on selective GM17 (Em: 2.5 μg mL^−1^) or MRS (Em: 5 μg mL^−1^) agar plates.

Tissue biopsies were fixed, processed, and analyzed by transmission electron microscopy (TEM) as described previously [22]. For this purpose, blood was centrifuged (620× g for 20 min 4 °C), to collect the cells and the pellet was embedded in an agarose matrix before fixing in paraformaldehyde and glutaraldehyde. 

### 2.6. Statistics Analysis: Sample Size Calculation

The recommended number of animals included in this trial was determined using the G*Power 3.1.9.2 program [23], and previous results obtained after oral administration of a genetically labeled *Enterococcus faecium* strain to pregnant mice [24]. The minimum value for the frequency of detection of the *lux*-labeled strain in the experimental group was estimated to be 80%, while it was not expected to be found in the control group. The minimum sample size was estimated to be 12 animals, using a 1:1 allocation ratio, and considering a one-tailed test, a 5% alpha level, and a statistical power of 90% to demonstrate a significant difference. Since one mouse in the control group was lost (a mouse that did not become pregnant) and the real proportion of animals containing *lux*-labeled *Lactococcus* in the experimental group was higher than 80%, a post hoc analysis was performed. This analysis confirmed that the achieved power was higher (>99%) than the value that had been selected initially for the calculation.

## 3. Results

### 3.1. Transformation of E. coli and LAB Strains with pMG36e::luxAB and pMG36e::luxABCDE

**Figure 1:**
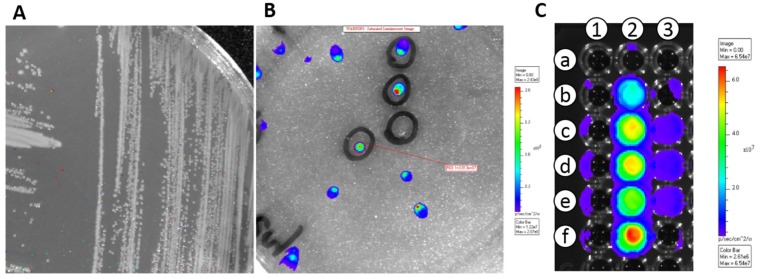
Transformation of *E. coli* with the *lux* operon. (**A**) LB agar plate showing non-transformed *E. coli* cells (negative control). (**B**) LB agar plate showing *E. coli* cells transformed with pMG36e::*luxABCDE*. (**C**) Microtiter plate showing *E. coli* cells transformed with pMG36e::*luxABCDE*; Column 1: non-inoculated LB medium (negative control); Column 2: files b to f: serial dilutions of *E. coli* cells transformed with pMG36e::*luxABCDE*.

Transformation of competent *E. coli* cells with plasmid pMG36e::*luxAB* or pMG36e::*luxABCDE* led to bacterial grow in selective conditions. Some colonies showed bioluminescence both in broth (RLU > 1500) and agar plates and, therefore, were selected for further studies (Figure 1). The presence of the *lux* operon in the transformed cells was also confirmed by PCR amplification of *lux* operon-specific fragments.

**Figure 2:**
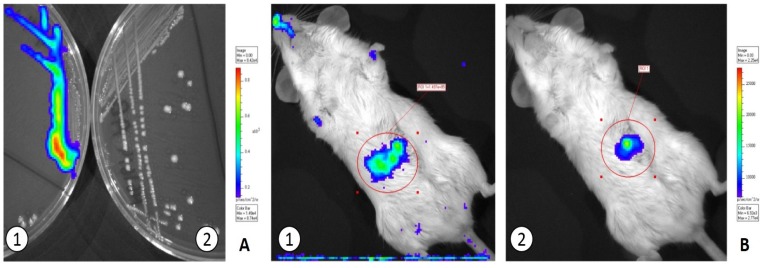
In vitro and in vivo detection of *L. lactis* MG1614 transformed with pMG36::*luxABCDE*. (**A**) GM17 agar plate with transformed (left) and non-transformed (right) *L. lactis* MG1614 cells. (**B**) Mouse immediately (left) and 20 min (right) after being fed with *L. lactis* pMG36e::*luxABCDE*.

Similarly, the successful transformation of *L. lactis* MG1614 with either pMG36e::*luxAB* or pMG36e::*luxABCDE* and that of *L. salivarius* PS2 with pMG36e::*luxAB* was confirmed by bioluminescence and PCR (Figure 2). However, the transformation of *L. salivarius* PS2 with pMG36e::*luxABCDE* was not possible after several electroporation attempts. The addition of d-luciferin Firefly (Xenogen) to the growth medium (150 μg/mL) was required in order to generate and detect bioluminescence by *L. salivarius* PS2 containing only the *luxAB* genes.

The stability of *L. salivarius* PS2 transformed with pMG36e::*luxAB* was measured during~100 generations under selective (Em-supplemented) and non-selective conditions. The growth of the transformed strain in selective medium was ~1 log_10_ cycle higher in comparison to that observed in the non-selective one. Such difference remained constant along time, indicating that recombinant plasmid was stable in *L. salivarius* PS2.

### 3.2. In Vivo Translocation Model

A strong bioluminiscence signal was detected in the stomach of mice during the first 20 min after oral administration of the transformed strains (Figure 2). The bacteria gradually entered (and became diluted) in the gut compartment and, as a consequence, bioluminescence was lost ~1 h after their administration.

**Figure 3:**
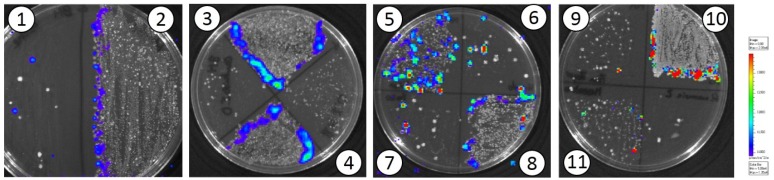
Isolation of transformed *L. lactis*MG1614 cells on GM17 agar plates from different maternal biological samples and organ biopsies: 1: milk; 2: feces; 3: small intestine; 4: large intestine; 5: kidney; 6: liver; 7: spleen; 8: stomach; 9: Peyer’s patch; 10: urine; and 11: mammary gland.

In relation to recombinant *L. lactis* (pMG36::e*luxABCDE*), mice were sacrificed at the end of the assay and biological samples (milk, urine, and feces) and biopsies of different organs and tissues were collected, homogenized (when required), and seeded on plates of agar GM17 supplemented with Em (2.5 μg mL^−1^). After incubation, bioluminescent *L. lactis* colonies could be isolated from the samples of milk, urine, and feces and from the biopsies of mammary gland, liver, kidney, intestine, stomach, Peyer patches, spleen, and uterus (Figure 3).

With respect to recombinant *L. salivarius* (pMG36::e*luxAB*), the four mice that received the strain and one from the control group were sacrificed within one day after delivery. Feces and biopsies from small intestine, cecum, mammary gland, spleen and mesenteric lymph nodes were collected, homogenized (when required), and seeded on MRS and on MRS supplemented with Em(5 μg mL^−1^) agar plates. PCR analyses were also carried out to detect the presence of the *luxAB* genes in the biological samples. The counts obtained from the different samples analyzed in this study are shown in Table 2. Bacterial growth was observed in the fecal samples from all the animals. As expected, fecal bacterial counts (expressed as cfu/g) were 3–4 log_10_ cycles higher in MRS (non-selective medium) than in Em-supplemented MRS (selective medium) plates. The *luxAB* fragment could be detected by PCR from feces of the four mice that received the recombinant strain but not from those collected from the control animal. In addition, the strain could be isolated and PCR-detected from mammary biopsies of the pregnant mice but not from those collected from the non-pregnant mice. Bacterial counts in the mammary samples were notably lower than fecal counts obtained from the same media and from the same animal (Table 2). 

**Figure 4:**
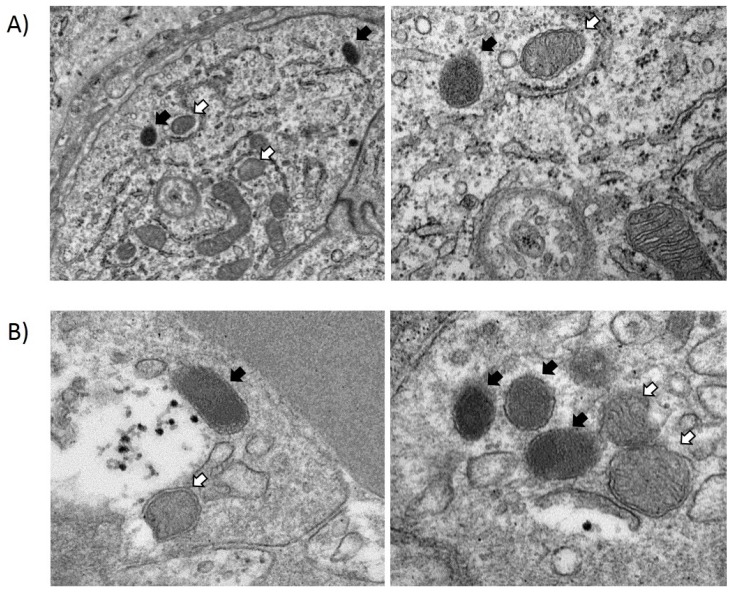
Transmission electron microscopy (TEM) images. Bacteria (black arrows) and mitochondria (white arrows) present in samples from a mesenteric lymph node (**A**) and spleen (**B**).

The *luxAB* fragment could not be detected either in spleen or in mesenteric lymph node samples. However, bacteria were observed in mesenteric lymph nodes (Figure 4A) and spleen (Figure 4B) by TEM image analysis. Using such a technique, bacterial cells appeared as a double membrane surrounding a dense content (chromatin) (Figure 4). Similar structures were detected in mammary gland biopsies. 

## 4. Discussion

In this work, two LAB strains (*L. lactis* MG1614 and *L. salivarius* PS2) were genetically modified to harbor genes belonging to the *lux* operon. This strain-labeling method constitutes an excellent tool for in vivo whole-body detection of bacteria in animal models [25]. Its advantages include (a) a very sensitive and rapid detection of luciferase activity, (b) a luminescent response that is linearly dependent on the amount of luciferase, and (c) the possibility of using real-time non-invasive techniques when combined with low-light imaging CCD cameras [25,26].

The recombinant plasmid pMG36e::*luxABCDE* was successfully transformed into *L. lactis* MG1614, but it could not be successfully electroporated into *L. salivarius* PS2. The large size of the plasmid (>10 kb) and the fact that *L. salivarius* PS2 is a wild strain may explain our inability to obtain transformants. Fortunately, *L. salivarius* PS2 cells were transformed with the smaller pMG36e::*luxAB* plasmid. In the absence of the *luxCDE* genes, strain-specific bioluminescence can still be detected provided that d-luciferin is added to the growth medium.

Both strains could be isolated (and the *lux* genes detected by PCR) from either milk or mammary gland biopsies after their oral administration to pregnant mice. While some may argue that their presence in milk might be the result of superficial fecal contamination in mice, such a route can hardly explain their isolation and detection from mammary biopsies. In a previous work, oral administration of *L. salivarius* PS2 to pregnant women led to the presence of the strain in the milk of some of the women after delivery [16]. It must be noted that, in contrast to mice, fecal contamination of human milk is highly improbable and, in fact, enterobacteria are usually absent in milk samples collected by manual expression from healthy women [27]. Our results reinforce the hypothesis that, at least, some members of the milk microbiota may arise from the digestive tract of the mother.

In the last years, different studies have shown that milk from healthy women contain bacteria that are subsequently transferred to the infant gut [1,28,29,30,31,32,33,34,35,36]. The detection of cells and/or DNA belonging to anaerobic species that are related to the adult gut environment (*Blautia*, *Bifidobacterium*, *Bacteroides*, *Parabacteroides*, *Clostridium*, *Collinsella, Coprococcus*, *Faecalibacterium*, *Roseburia*, *Subdoligranulum,* or *Veillonella*) has ledtothe hypothesisthat some of the human milk bacteria may originate in the maternal digestive tract (mouth, gastrointestinal tract) and reach the mammary gland through an endogenous route [3,4,31,33,37]. 

So far, culture-independent studies have not provided information on microbial viability and have not allowed strain level discrimination. A second caveat of molecular methods is the low amount of DNA typically extracted from milk samples, making the relative proportion of contaminant DNA from sample manipulation and from DNA extraction reagents more important than when analyzing other biological samples, such as feces [33,38]. However, the role of breastfeeding in the vertical mother-to-infant transfer of specific bacterial strains (including bifidobacteria and lactic acid bacteria strains) has already been demonstrated [6,16,31,33,34,35,38,39,40,41,42,43,44,45]. Some studies have revealed the ability of certain gut bacteria to spread to extra-digestive locations in healthy hosts [46,47,48,49,50], while others (including in vitro, animal, and human studies) have shown that physiological bacterial translocation during late pregnancy has a scientifically plausible basis and may involve complex interactions between microbes, immune cells, and gut epithelial cells [4,5,6,16,47,51,52,53,54,55,56].

Gut bacteria translocation has usually been associated with pathogenic conditions [57,58,59,60], but a low rate of bacterial translocation (involving *Bacteroides*, lactobacilli, bifidobacteria, or enterococci) occurs in healthy hosts [12,60,61,62,63,64,65,66] and may be associated to physiological immunomodulation [4,67,68]. Some bacterial strains seems to specifically mediate their own translocation without collateral translocation of other bacteria from the host digestive tract [12,69]. Many transient anatomical and physiological changes that occur during pregnancy and lactation may favor an increased bacterial translocation during such periods [1,62,70,71,72] and have been reviewed in [37]. 

At present, it is widely accepted that microbes are present in diverse organs and tissues previously thought to be sterile environments, including tumors [12]. Oral administration of a probiotic *Bifidobacterium breve* strain harboring a *lux*-expressing plasmid to mice bearing subcutaneous tumors led to its presence in tumors at levels similar to intravenous administration [12]. Similarly, another probiotic strain (*E. coli* Nissle 1917) robustly colonized tumor tissue in rodent models of liver metastasis after oral delivery but did not colonize healthy organs or fibrotic liver tissue [69]. The authors suggested that oral delivery could lead to the preferential colonization of liver tumors by allowing probiotics to follow physiological blood flow patterns, wherein the venous outflow from the gut is directed to the liver via the hepatic portal vein. This phenomenon has been attributed to at least three different factors: (a) suppressed immune surveillance within the tumor, (b) tumor vascularization, and (c) increased availability of nutrients in the necrotic tumor core [73,74,75,76]. It has been proposed that the entry, survival, and replication of bacteria in tumors depends on the vascularization and immune-privileged nature of solid tumors, which may provide a suitable microenvironment for a small spectrum of bacterial species [74]. The nutrient-rich environment may also play an important role since tumors can support the growth of auxotrophic *S. typhimurium* strains [76,77,78]. Interestingly, the same factors are also present in the mammalian glands during late pregnancy and may explain the selective tropism or homing effect that the mammary gland seems to exert on some maternal bacterial species during this life stage: (a) there is a physiological immunodepression state in order to tolerate the fetus; (b) as stated above, there is a formidable angiogenesis process; and (c) pre-colostrum starts to fill the mammary duct during the last third of pregnancy providing a rich nutrient environment for bacteria.

Further studies are required to elucidate the mechanisms by which some bacterial strains may translocate physiologically in certain hosts or life stages. The existence of such bacterial oral- and entero-mammary pathways would provide new opportunities for manipulating an altered maternal-fetal microbiota, reducing the risk of preterm birth or infant diseases. 

## 5. Conclusions

Human milk is one of the main sources for the vertical mother-to-infant transmission of bacteria in early life. Although the origin of the bacteria naturally present in this biological fluid can be diverse, the results of this study confirm that there may be a physiological translocation of certain bacterial strains from the maternal digestive tract to the mammary gland and milk.

## Figures and Tables

**Table 1:** Primers used in this study for the Polymerase Chain Reaction (PCR) detection of *lux* genes.

Name	Sequence (5′-3′)	Reference or Source
XAF1	CCC CGA GCT CAT GAA GCA AGA GGA GGA CTC TCT ATG	Modified from [17]
XBR1	GGC CCC GGG TTA GGT ATA TTC CAT GTG GTA C	Modified from [17]
XAF1	CCC CGA GCT CAT GAA GCA AGA GGA GGA CTC TCT ATG	Modified from [17]
XER2	GGC GGC GTC GAC TTA ACT ATC AAA CGC TTC GGT TA	Modified from [17]
lux1280	ACG CCG CAG GAA TGT ATT GA	This study
lux1732	TAT GGC GAC AGG ATG ATG AG	This study
lux4807	GTC AAT GAA CGC CGA ATG AG	This study
lux5068	GTC ACT ACT GTC AGG CAC AC	This study

**Table 2:** *L. salivarius* PS2 counts (log_10_ cfu/g) in the biological samples collected from mice (1 and 2: non-pregnant mice; 3 and 4: pregnant mice) that were fed with *L. salivarius* PS2 cells transformed with plasmid pMG36e::*luxAB.*

Sample	Mice	Growth Medium	
		MRS	MRS-Em^a^
Feces	1	na	5.92
	2	7.96	4.72
	3	7.54	4.26
	4	7.96	3.48
Small intestine	1	4.80	3.00
	2	4.64	nd
	3	4.96	nd
	4	na	na
Large intestine	1	6.65	5.54
	2	na	na
	3	6.48	2.70
	4	6.23	4.56
Spleen	1	nd	nd
	2	nd	nd
	3	nd	nd
	4	3.65	3.90
Mammary gland tissue	1	nd	nd
	2	nd	nd
	3	4.98	3.30
	4	4.98	3.00

^a.^ MRS supplemented with erythromycin; na: sample not available; nd: not detected.
